# Engineering proximal *vs.* distal heme–NO coordination *via* dinitrosyl dynamics: implications for NO sensor design†
†Electronic supplementary information (ESI) available: Structure factors and atomic coordinates have been deposited in the RCSB Protein Data Bank with accession codes; 5JT4, 5JLI, 5JP7, 5JRA, 5JVE, 5JUA, 5JSL, 5JS5. See DOI: 10.1039/c6sc04190f



**DOI:** 10.1039/c6sc04190f

**Published:** 2016-11-16

**Authors:** Demet Kekilli, Christine A. Petersen, David A. Pixton, Dlzar D. Ghafoor, Gaylany H. Abdullah, Florian S. N. Dworkowski, Michael T. Wilson, Derren J. Heyes, Samantha J. O. Hardman, Loretta M. Murphy, Richard W. Strange, Nigel S. Scrutton, Colin R. Andrew, Michael A. Hough

**Affiliations:** a School of Biological Sciences , University of Essex , Wivenhoe Park , Colchester , Essex CO4 3SQ , UK . Email: mahough@essex.ac.uk; b Department of Chemistry and Biochemistry , Eastern Oregon University , La Grande , Oregon 97850 , USA . Email: candrew@eou.edu; c Faculty of Science and Education Science , University of Sulaimani , Sulaymaniyah , Iraq; d Medical Research Center , Hawler Medical University , Erbil , Iraq; e Swiss Light Source , Paul Scherrer Institute , Villigen PSI , CH-5232 , Switzerland; f Manchester Institute of Biotechnology , 131 Princess Street , Manchester M1 7DN , UK; g School of Chemistry , Bangor University , Bangor , Gwynedd , Wales LL57 2UW , UK; h Molecular Biophysics Group , Institute of Integrative Biology , Faculty of Health and Life Sciences , University of Liverpool , Liverpool , L69 7ZB , UK

## Abstract

Proximal *vs.* distal heme–NO coordination is a novel strategy for selective gas response in heme-based NO-sensors. In the case of *Alcaligenes xylosoxidans* cytochrome c′ (AXCP), formation of a transient distal 6cNO complex is followed by scission of the *trans* Fe–His bond and conversion to a proximal 5cNO product *via* a putative dinitrosyl species. Here we show that replacement of the AXCP distal Leu16 residue with smaller or similar sized residues (Ala, Val or Ile) traps the distal 6cNO complex, whereas Leu or Phe residues lead to a proximal 5cNO product with a transient or non-detectable distal 6cNO precursor. Crystallographic, spectroscopic, and kinetic measurements of 6cNO AXCP complexes show that increased distal steric hindrance leads to distortion of the Fe–N–O angle and flipping of the heme 7-propionate. However, it is the kinetic parameters of the distal NO ligand that determine whether 6cNO or proximal 5cNO end products are formed. Our data support a ‘balance of affinities’ mechanism in which proximal 5cNO coordination depends on relatively rapid release of the distal NO from the dinitrosyl precursor. This mechanism, which is applicable to other proteins that form transient dinitrosyls, represents a novel strategy for 5cNO formation that does not rely on an inherently weak Fe–His bond. Our data suggest a general means of engineering selective gas response into biologically-derived gas sensors in synthetic biology.

## Abstract 2

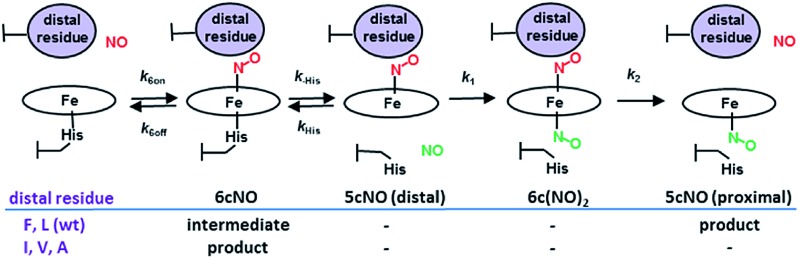
Distal *versus* proximal binding of nitric oxide to haem is controlled *via* a ‘balance of affinities’ kinetic mechanism.

The molecular mechanisms that underpin heme protein discrimination between diatomic gas ligands (NO, CO and O_2_) are of fundamental importance in cellular respiration, signalling and toxicity defence and are thus of wide relevance. Understanding the means by which heme proteins are able to produce selectivity and discrimination in their binding of gases is vital for the effective design or adaptation of heme-based sensors for biotechnology. Many penta-coordinate heme proteins, for example the archetypal myoglobin, simply bind gases to their vacant sixth coordination position at the distal heme face. However, in an increasing number of proteins it has been shown that the key signalling molecule nitric oxide (NO) forms a five-coordinate complex with NO (5cNO) bound to the proximal face of the heme. First identified in cytochrome c′ from *Alcaligenes xylosoxidans* (AXCP),^1^ proximal NO binding has recently been confirmed also to occur in the NO-activation mechanism of the eukaryotic NO sensor soluble guanylate cyclase (sGC),^2,3^ bacterial heme nitric oxide/oxygen binding (H-NOX) gas sensors,^4–6^ and the pro-apoptotic cytochrome c/cardiolipin complex.^7^ The factors that control distal *vs.* proximal heme–NO coordination in these proteins are therefore of particular interest.

Cytochromes c′ occur in methanotrophic, denitrifying and photosynthetic bacteria and have proposed roles in protection against nitrosative stress, NO trafficking during denitrification or pathogen defence.^8^ All cytochromes c′ have a 4 α-helix bundle structure containing a heme centre with a solvent exposed proximal His ligand and a buried hydrophobic distal pocket with a non-coordinated residue (Leu or Phe, rarely Met or Tyr) in a position to exert steric influence on the binding of diatomic gases. Unusually, cytochromes c′ are able to utilize both heme faces (distal and proximal) as a means of discriminating NO from other diatomic gases. Studies of heme–NO-binding in AXCP reveal a multi-step dissociative mechanism in which formation of a distal six-coordinate heme–nitrosyl (6cNO) complex is followed by scission of the *trans* Fe–His bond and conversion to a proximal 5cNO product *via* a putative dinitrosyl species (Scheme 1).^9^ While such dinitrosyl complexes are transient in proteins, they have also been experimentally characterised in small molecule porphyrin complexes.^10^

**Scheme 1:**
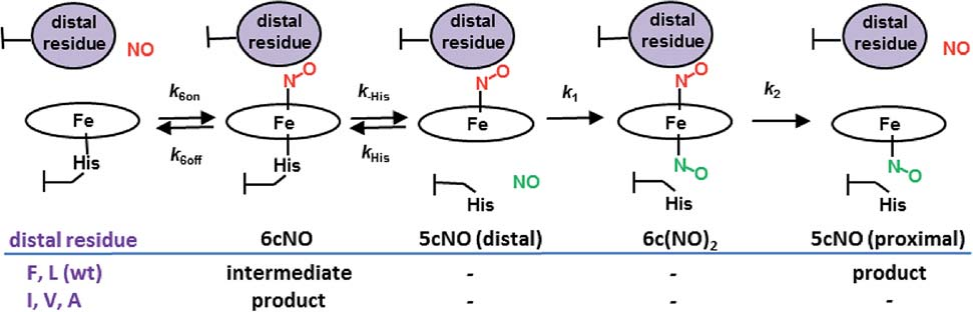
AXCP heme–NO binding mechanism and the effect of distal Leu16 mutations on observed intermediates and products.

In heme proteins, 5cNO formation has been traditionally linked to an inherently weak Fe–His bond, which upon distal NO binding, facilitates His ligand release *via* a negative *trans* effect. However, cytochromes c′ are unusual because they form 5cNO complexes despite relatively strong Fe–His bonds, with *ν*(Fe–His) frequencies (∼230 cm^–1^) significantly higher than the cut off limit of ∼216 cm^–1^ beyond which heme proteins are predicted to remain in the 6cNO state.^11^ Moreover, the fact that the L16A variant of AXCP (which forms only a distal 6cNO state) has a similar *ν*(Fe–His) frequency to that of wt AXCP (which forms a proximal 5cNO product) strongly suggests that distal 6cNO → proximal 5cNO conversion is governed by factors other than the Fe–His bond strength.^12^

Previous studies showed a greatly increased affinity for NO (also CO, O_2_) in the L16A variant of AXCP^12,13^ with a 6cNO distal complex being trapped and no proximal NO formation. In order to understand the factors controlling distal *versus* proximal NO coordination, we have examined NO binding to AXCP variants in which the occluding distal residue Leu16 is replaced with residues that are smaller (Ala, Val), of comparable size (Ile) or larger (Phe). The data are consistent with a ‘balance of affinities’ mechanism where the distal pocket occluding residue affects the kinetic parameters *k*_6on_ and *k*_6off_ for NO binding and release at the distal heme face. The ratio of distal *vs.* proximal face affinities determines which NO dissociates from the transient dinitrosyl intermediate, leaving either a distal 6cNO complex (L16A, L16V, L16I) or a proximal 5cNO complex (wt AXCP, L16F). Our mechanism provides a novel route for 6cNO → 5cNO conversion that does not require an inherently weak Fe–His bond. It is the steric environment that underpins the reactivity differences between distal and proximal sites. The same balance of affinities mechanism (perhaps involving other structural properties) could operate in any naturally occurring or engineered heme-based NO sensor that generates a transient dinitrosyl species.

## Results and discussion

### Crystal structures and RR spectra of ferrous and ferrous NO proteins

**Fig. 1:**
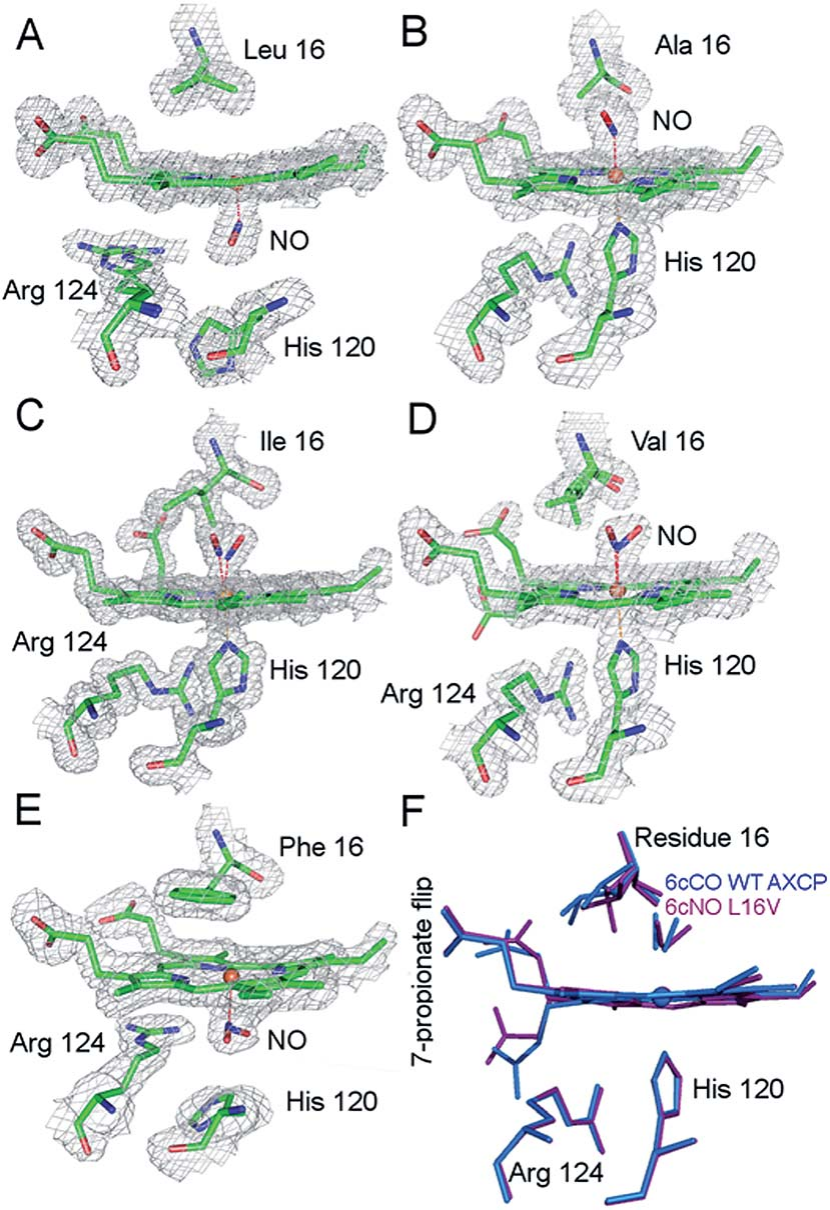
2*F*_o_ – *F*_c_ electron density maps of the ferrous–nitrosyl complexes in wt AXCP and distal variants (contoured at 1.0*σ*). (A) Proximal 5cNO complex in wt AXCP. (B) Distal 6cNO complex of L16A (C). Distal 6cNO complex of L16I with the presence of two NO conformers and one Ile16 conformation but the absence of the 7-propionate flip. (D) Distal 6cNO complex of L16V with the presence of two NO and Val16 conformers and a partial 7-propionate flip. (E) Proximal 5cNO complex in L16F. (F) Superposition of the distal 6cNO complex in the L16V variant (purple) and the distal 6cCO wt AXCP structure (blue). *F*_o_ – *F*_c_ omit maps for NO complexes are shown in Fig. S5.†

All AXCP variants studied (L16A, V, I, and F) exhibit Fe(ii) heme absorption spectra similar to that of wt protein^14^ (Fig. S1a†). The *ν*(Fe–His) frequencies of Fe(ii) L16V (234 cm^–1^) and Fe(ii) L16I (235 cm^–1^) from room-temperature resonance Raman (RR) spectra (Fig. S1, Table S1†) resemble those of L16A (230 cm^–1^) and wt AXCP (231 cm^–1^) and signify a relatively strong proximal bond. On addition of excess NO, the L16F variant forms a 5cNO product (*λ*_max_ ∼ 395 nm) similar to that of wt AXCP while the L16A, L16V and L16I variants instead form only a 6cNO product (*λ*_max_ ∼ 415 nm) (Fig. S2†). Crystal structures were determined for the ferrous and ferrous nitrosyl complexes of the variants, with redox and ligand states validated using *in situ*, on axis single crystal resonance Raman (SCRR) spectroscopy (Tables 1, S2 & S3, Fig. 1 and S3 & S4†) using methods previously described.^14,15^ All of the tertiary structures are similar to wt AXCP and only salient points concerning the heme region are discussed here. Ferrous structures show the different residues at position 16 to lie over the heme in a similar manner to that of Leu16 in wt AXCP (Fig. S4†) with the exception of L16A where the side chain is essentially truncated. While the structures of ferrous L16V, L16I, and wt AXCP have empty distal sites, in L16A a well-defined water ligand (not observed in solution RR spectra) is present at a distance of 2.17 Å (Fig. S4†).

**Table 1:** Structural parameters from crystal structures of ferrous heme–NO complexes of wt AXCP and variants^*a*^ ^,^ ^*b*^

Res	Heme–NO	Resolution (Å)	Fe–His (Å)	Fe–N (NO)	Fe–N–O (°)
Ala	Distal 6cNO	1.55	2.16	1.81	135
Val	Distal 6cNO	1.38	2.18	1.85/1.69	127/132
Ile	Distal 6cNO	1.13	2.12(0.01)	1.66(0.04)/1.87(0.03)	139(3)/136(4)
Leu^a^	Proximal 5cNO	1.26	—	1.84	142
Phe	Proximal 5cNO	1.70	—	2.18/1.98	118/118

^*a*^All values are from this work, except, ^a^.^15^

^*b*^Values in parentheses are estimated standard deviations from inversion of the least squares matrix in SHELXL (for structures at 1.25 Å resolution or better). The lengthening of the Fe–His bonds of 6cNO species relative to their ferrous state (Table S3) is indicative of a negative *trans* effect.

In sharp contrast to the proximal 5cNO product formed by wt AXCP (Fig. 1a), the structure of ferrous nitrosyl L16A (Fig. 1b and S5,† Table 1) confirms the presence of a distal 6cNO complex as suggested by spectroscopic data.^12^ The distal NO ligand at full occupancy does not form any hydrogen bonds to protein residues and the Fe–N–O angle is 135° with no indication of steric conflict between the Ala16 residue and NO. Substitution of the occluding Leu16 residue in the distal pocket by the smaller Ala thus results in NO binding essentially without steric effects from the protein environment and with Fe–N–O geometry similar to that of {FeNO}^7^ model complexes. Consistent with the removal of distal steric hindrance, the Leu16 → Ala mutation is associated with a ∼100-fold increase in *k*_6on_ (*vide infra* and ref. 12 and 13).

Compared to L16A, the L16I AXCP variant experiences greater steric hindrance towards distal NO binding (Fig. 1c and S5†). The crystal structure of the 6cNO L16I complex shows two orientations of NO, oriented towards Met19 and Pro55 respectively. The Fe–N–O angles for these are very similar (136° and 139°) and only one conformer of Ile16 is present. Ile16 undergoes a modest shift in position (∼0.9 Å) upon binding of NO while maintaining a similar rotamer.

In this structure, the heme 7-propionate is not flipped to the proximal pocket (as observed in the 6c–CO complex of wt AXCP) but does have an altered conformation. Relative to L16I, the L16V variant exhibits more extensive rearrangements upon 6cNO formation. Although smaller than Ile, the orientation of the Val side chain in the L16V variant brings it closer to the heme propionates (Fig. 1d and S5†) and there is a small main chain shift of the Val away from Met19.

Two NO orientations with partial occupancies are also present in L16V. In one of these, NO is positioned towards the side chain of Met19 and the Fe–N–O angle is 127°. In the second conformation, NO is oriented towards Trp56 and the Fe–N–O angle is 132°. In the 132° conformer (but not the 127° conformer), the Val16 conformation has undergone a ∼180° rotation, causing it to occupy a position where it would provoke a steric clash with the orientation of the distal heme 7-propionate (3.37 Å compared to 4.20 Å) in the 5c ferrous structure, causing the propionate to flip towards the proximal pocket (Fig. 1d and S5†). These structural rearrangements are analogous to those recently observed for the 6cCO complex of wt AXCP, in which a near-linear Fe–C–O geometry forces a 120° rotation of the Leu16 C_α_–C_β_ bond together with a distal to proximal flip of the 7-propionate. A second conformer with a bent Fe–C–O (158°) is associated with smaller movements of Leu16 and no propionate flip.^13^ The reason why a Fe–C–O angle of 158° does not lead to a propionate flip, whereas a more compressed Fe–N–O angle of 132° does may indicate that the orientation of the Fe–X–O unit (not just the bond angle) is important.

A superposition of the wt AXCP 6cCO structure with the L16V 6cNO structure is given in (Fig. 1f). While the structure of the transient 6cNO complex of wt AXCP has yet to be determined, the structural rearrangements observed upon L16V 6cNO and wt 6cCO formation suggest that distal NO binding to wt AXCP is also likely to involve significant rotation of the Leu16 residue and a proximal flip of the 7-propionate. Further insights into the 6cNO structure are provided by RR spectroscopy (*vide infra*). Finally, the crystal structure of the L16F variant shows two equally occupied proximal 5cNO binding modes (Figs. 1e, S5, Table 1), similar to the proximal complex previously observed for the wt AXCP end product, and is not described in detail here. Kinetic data confirm that this variant has a high degree of distal steric constraint that destabilizes distal heme–NO binding (*vide infra*).

**Fig. 2:**
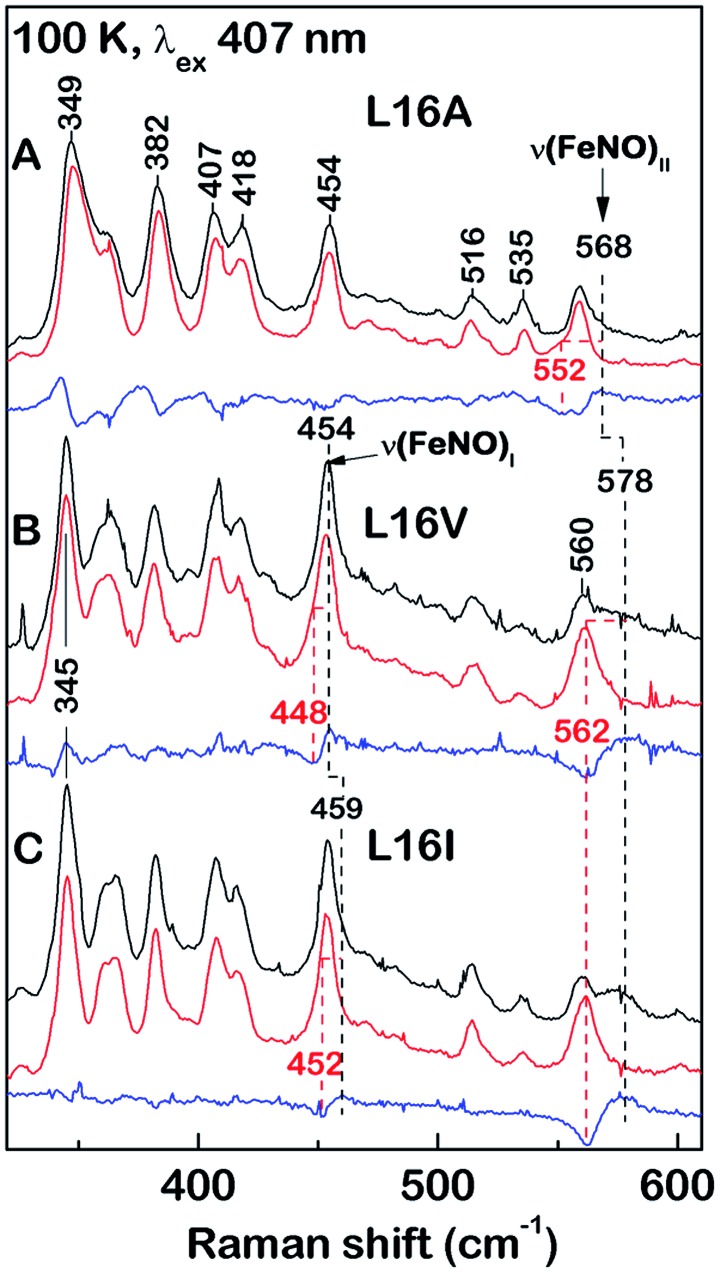
Low-frequency RR spectra of 6cNO AXCP solutions (100 K) obtained with 406.7 nm excitation: (A) L16A, (B) L16V, and (C) L16I proteins prepared with ^14^NO (black) and ^15^NO (red). Isotope difference spectra (blue) identify the *ν*(FeNO)_I_ and *ν*(FeNO)_II_ vibrations. The L16A *ν*(FeNO)_I_ frequency is identified from a larger isotope shift with ^15^N^18^O (Fig. S8†).

RR spectra of 6cNO AXCP complexes yield complementary structural information on the heme–NO environment in solution (Fig. 2 and S6–S8†). Porphyrin marker vibrations of frozen solutions (100 K) have frequencies typical of 6cNO heme (Fig. S6 & S7,† Table 2), and are similar (±5 cm^–1^) to those obtained from single crystals (Fig. S3,† Table 2).

**Table 2:** Heme–NO vibrational frequencies (cm^–1^) of 6cNO heme proteins^*a*^

6cNO protein		Temp	*ν*_4_	*ν*_3_	*ν*_2_	*ν*_10_	*ν*(FeNO)_I_	*ν*(FeNO)_II_	*ν*(NO)	Ref.
AXCP	(L16A)	rt	1373	1500	1593	1631	454	563	1630	tw
		100 K	1373	1501	1595	1635	454	568	1631	tw
		100 K	*1372*	*1501*	*1592*	*1632*				tw
	(L16V)	100 K	1374	1501	1595	1634	454	578	1626	tw
		100 K	*1377*		*1597*	1632				tw
	(L16I)	100 K	1374	1501	1595	1634	459	578	1621	tw
		100 K	*1372*		*1592*					tw
	(wt)	rt							1625^b^	16
		90 K	1375	1504	1596	1638		579	1624	17
RCCP	(wt)	rt	1375	1503	1593	1635	458	562		18
		90 K	1377	1506	1598	1640	460	569	1624	18
Mb (sw)	(H64L)	293 K						560	1635	19

^*a*^Frequencies are from RR spectra of protein solutions at pH 7.0, or from single crystals at pH 7.5 (data in italics) except for ^b^stopped-flow FTIR data at pD 9.4. Abbreviations: rt; room temperature, tw; this work, sw; sperm whale.

The *ν*(N–O) stretching frequencies of L16A (1630 cm^–1^), L16V, (1626 cm^–1^), and L16I (1621 cm^–1^) are identified from their ∼25 cm^–1^ downshifts with ^15^NO (Fig. S6†) or a ∼70 cm^–1^ downshift with ^15^N^18^O (Fig. S7†). Two bands with mixed Fe–NO stretching/bending character are also evident in the ∼450–460 cm^–1^ and ∼560–580 cm^–1^ regions, denoted *ν*(FeNO)_I_ and *ν*(FeNO)_II_ respectively (Fig. 2). Although vibrational assignments of 6cNO complexes have been controversial,^20–22^ recent nuclear resonance vibrational spectroscopy (NRVS) studies point to *ν*(FeNO)_II_ as the predominant Fe–N–O bend and *ν*(FeNO)_I_ (not always observed in RR spectra) as the predominant Fe–NO stretch.^23–25^ The *ν*(FeNO)_II_ modes of L16A (568 cm^–1^), L16V (578 cm^–1^), and L16I (578 cm^–1^) are readily identified from their ∼16 cm^–1^ downshifts with ^15^NO (Fig. 2) or ∼20 cm^–1^ downshift with ^15^N^18^O (Fig. S8†), while the relatively weak *ν*(FeNO)_I_ bands of L16A (454 cm^–1^), L16V (454 cm^–1^), and L16I (459 cm^–1^) – obscured by an overlapping porphyrin mode – are identified from ∼6 cm^–1^ downshifts with ^15^NO (Fig. 2) or a ∼10 cm^–1^ downshift with ^15^N^18^O (Fig. S8†). As previously observed for *Rhodobacter capsulatus* cytochrome c′ (RCCP),^18^ 6cNO RR frequencies are sensitive to sample temperature (Table 2).

Since all of the 6cNO AXCP variants have hydrophobic distal pockets (Fig. 1), it appears that the variations in RR frequencies arise from steric rather than electrostatic effects. DFT calculations by Spiro and co-workers predict that compression of the Fe–N–O angle below ∼140° should lower the *ν*(N–O) frequency,^23^ in agreement with our crystallographic and RR data. On the other hand, decreasing the Fe–N–O angle is also predicted to weaken the Fe–NO bond, whereas we observe that both *ν*(FeNO)_I_ and *ν*(FeNO)_II_ are at similar or higher frequencies in sterically constrained sites (Table 2). This discrepancy may reflect the difficulty of modelling the angular dependence of these mixed vibrational modes.^26^

We also note that correlations between spectroscopic and structural data could be affected by conformational differences between the crystalline and solution state. For example, the L16V and L16I structures exhibit multiple Fe–N–O conformers, whereas there is no evidence for multiple sets of heme–NO RR bands in solution. Nevertheless, comparison of the present (100 K) RR data with previous measurements on the frozen wt 6cNO AXCP intermediate reveals the influence of distal steric constraints on heme–NO vibrational frequencies. Most notably, the sterically constrained L16V and L16I 6cNO complexes exhibit a ∼10 cm^–1^ upshift in *ν*(FeNO)_II_ and a ∼5–10 cm^–1^ downshift in *ν*(NO) frequencies relative to L16A. Importantly, the RR frequencies of the L16V and -I variants resemble those of the transient wt 6cNO complex, implying that the structures of the heme–NO chromophores are similar (despite differences in 6cNO stability). This suggests that distortion of the heme–NO unit is not the ultimate determinant of distal 6cNO → proximal 5cNO conversion. Instead, our studies suggest that distal 6cNO *vs.* proximal 5cNO formation is determined by the kinetic properties of the distal and proximal heme faces (*vide infra*).

### Residue 16 profoundly affects the kinetics of NO binding and rebinding

**Fig. 3:**
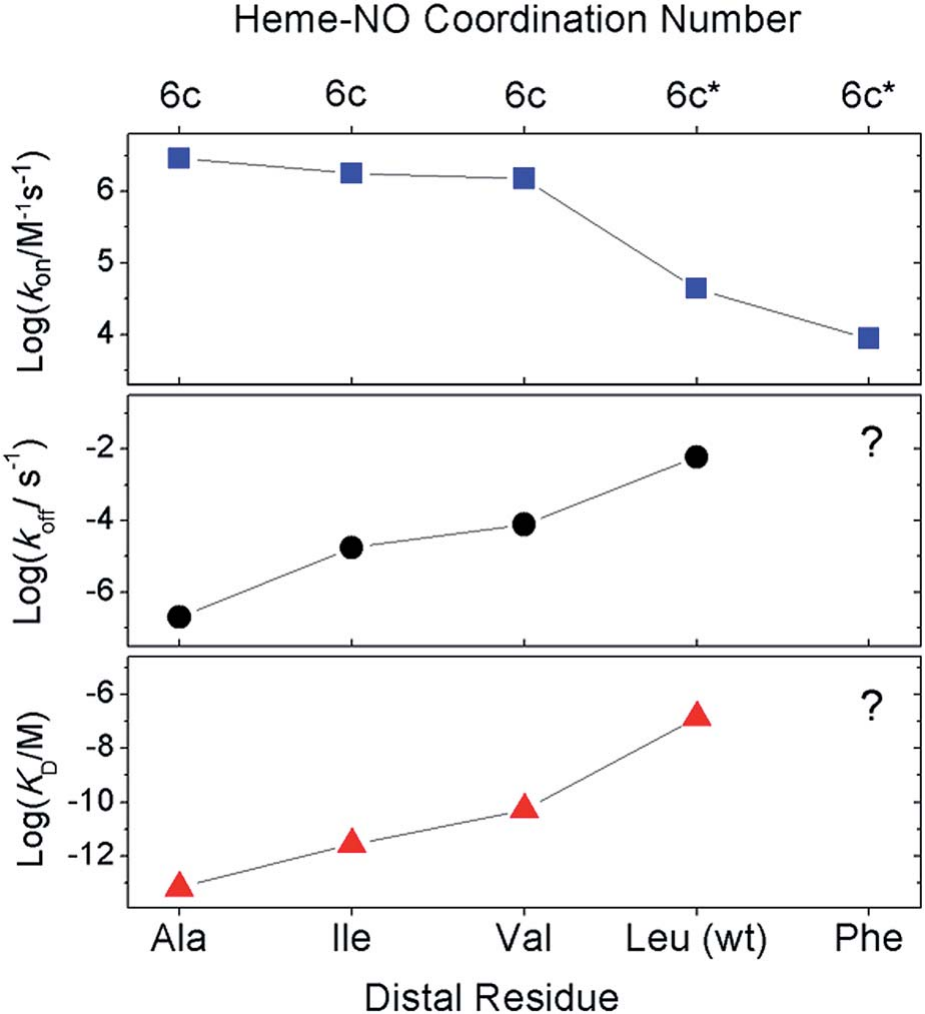
Effect of distal mutations on the values of *k*_6on_ (blue squares), *k*_6off_ (black circles), and *K*_D_ (red triangles) of 6cNO AXCP complexes. Asterisks denote transient 6cNO precursors to proximal 5cNO products.

We used stopped-flow optical spectroscopy to understand how the distal L16 mutations and the consequent structural changes in the distal pocket perturbed reactivity. Kinetic constants obtained are summarized in Table 3 and Fig. 3. As previously observed,^9,17^ wt AXCP undergoes an initial distal NO-binding event to form an observable 6cNO intermediate (*k*_6on_) which subsequently converts to a proximal 5cNO product (*k*_6-5_) (Fig. S9†). The L16F variant forms a proximal 5cNO complex in a similar manner to wt, albeit with changes to the kinetic parameters (Fig. S9,† Table 3). By contrast, the L16V, -A, and -I variants form stable distal 6cNO products (Fig. S10†) without the ‘distal-to-proximal’ conversion exhibited by wt and L16F AXCP (Fig. S9†).

**Table 3:** Kinetic and thermodynamic constants for 6cNO AXCP complexes in pH 8.9 buffer solutions at 25 °C^*a*^

Res16	Distal 6cNO	Proximal 5cNO	*k*_6on_ (M^–1^ s^–1^)	*k*_6off_ (s^–1^)	*K*_D_ (M)	*k*_6-5_ (M^–1^ s^–1^)	Reference
Ala	Product	no	2.9 × 10^6^	2.0 × 10^–7^	6.90 × 10^–14^	no	12
Val	Product	no	1.52 (±0.03) × 10^6^	7.80 × 10^–5^	5.13 × 10^–11^	no	tw
Ile	Product	no	1.79 (±0.15) × 10^6^	1.78 × 10^–5^	9.94 × 10^–12^	no	tw
Leu (wt)	Intermediate	Product	4.33 (±0.04) × 10^4^	6.0 × 10^–3^	1.40 × 10^–7^	1.14 (±0.04) × 10^4^	9
Phe	Intermediate	Product	8.88 (±0.37) × 10^3^	nd	nd	3.57 (±0.68) × 10^3^	tw

^*a*^Abbreviations: no; not observed, tw; this work, nd; not determined..

Previous kinetic data revealed that *k*_6on_ for L16A (2.9 × 10^6^ M^–1^ s^–1^) increased by two orders of magnitude relative to wt AXCP (4.33 ± 0.04 × 10^4^ M^–1^ s^–1^) (Table 3).^12,13^ Here we show that the *k*_6on_ values for L16V (1.52 ± 0.03 × 10^6^ M^–1^ s^–1^) and L16I (1.79 ± 0.15 × 10^6^ M^–1^ s^–1^) are midway between those of L16A and wt AXCP, consistent with intermediate residue sizes and steric constraints (Fig. 3, Table 3). The lower *k*_6on_ in L16V (Fig. S11†) relative to L16I and L16A is consistent with the more extensive structural rearrangements undergone by Val upon NO binding (*vide supra*). Rate constants for the release of NO (*k*_6off_) were also determined for L16V (7.80 × 10^–5^ s^–1^) and L16I (1.78 × 10^–5^ s^–1^) (Fig. S12†), and again these lie between the values for wt AXCP (6.0 × 10^–3^ s^–1^) and L16A (2.0 × 10^–7^ s^–1^) (Fig. 3, Table 3). However, whereas *k*_6on_ values decrease in response to steric hindrance, the values of *k*_6off_ progressively increase. Thus, the overall effect of distal steric constraints on *K*_D_ values (calculated from the *k*_6off_/*k*_6on_ ratio) is to lower the distal heme–NO affinity.

**Fig. 4:**
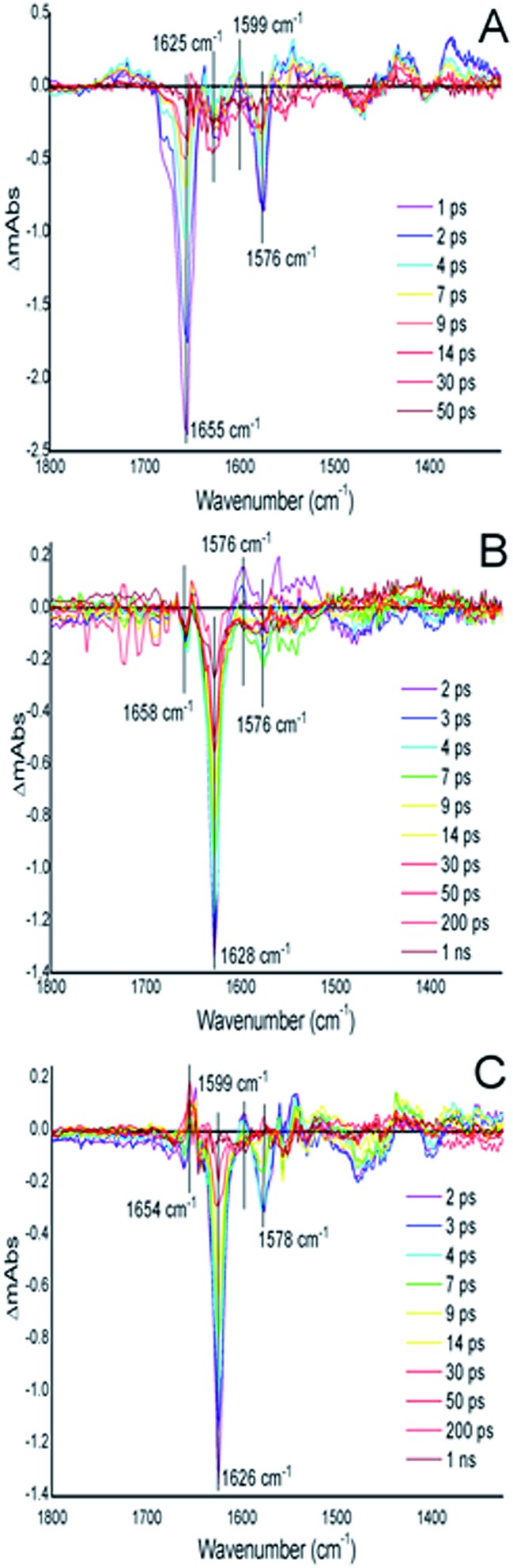
TRIR difference spectra for the wt AXCP, L16V and L16I variants. (A) wt AXCP difference spectra (1–50 ps) showing a ground signal bleach at 1655 cm^–1^ after laser photolysis corresponding to the cleavage of the 5c–NO bond followed by a return to the ground state (∼50 ps). (B) L16V variant difference spectra from 2–1000 ps showing a ground signal bleach at 1628 cm^–1^ and (C) L16I variant difference spectra (2–1000 ps) showing a ground signal bleach at 1626 cm^–1^ corresponding to the cleavage of the 6c–NO bond followed by a return to the ground state (∼1 ns).

In order to characterize the influence of the AXCP distal pocket structure on geminate recombination we carried out time-resolved infra-red (TRIR) experiments for NO rebinding following laser flash photolysis (Fig. 4 and S13†). In previous studies on wt AXCP, geminate rebinding of a population of distal 6cNO (generated by addition of sub-stoichiometric amounts of NO) was determined to occur with a time constant of 52 ps.^27^ Our TRIR measurements for the 6cNO complex of L16I and L16V indicate the rebinding of NO at the distal site with time constants of 6.8 ± 0.95 ps (L16I) and 7.4 ± 1.53 ps (L16V) with an additional slower phase with time constants of 52 ps (L16I) and 364 ps (L16V) (Fig. S13†). TRIR data for the L16A variant have been described previously.^28^ Faster and/or more complete geminate recombination in variants with smaller distal pocket residues could contribute to the observed changes in *k*_6off_ (NO). Future studies over extended timeframes will probe geminate NO rebinding to AXCP variants in more detail, including the influence of distal residue and heme 7-propionate rearrangements. Indeed, recent molecular dynamics simulations of geminate CO-rebinding in wt and L16A AXCP suggest that propionate conformation is a key determinant of distal AXCP-ligand affinity.^29^

### A balance of affinities mechanism for determining distal or proximal NO binding

Our data provide insights into the key question: why does wt AXCP undergo a distal 6cNO → proximal 5cNO conversion (involving Fe–His scission) whereas L16A, L16V and L16I variants remain 6cNO? Not only does AXCP not possess a weak Fe–His bond, the present study suggests that a distorted distal Fe–N–O geometry and/or flipping of the heme 7-propionate are not sufficient in themselves to drive proximal 5cNO conversion. Instead, kinetic trends show that proximal 5cNO formation occurs when the distal NO ligand of the 6cNO complex has a higher *k*_6off_ and a lower *k*_6on_ value (Fig. 3, Table 3).

Distal 6cNO → proximal 5cNO conversion involves breaking the Fe–His bond to form a putative distal 5cNO, followed by attack of a proximal NO to form a transient dinitrosyl and finally the release of the distal NO to generate a proximal 5cNO product (Scheme 1). We propose that the initial Fe–His bond scission by the *trans* effect is similar for wt and variant proteins, irrespective of the distal pocket occluding residue. Because of their relatively strong Fe–His bonds, we propose that the 6cNO species is in equilibrium with only a trace amount of distal 5cNO (below detection limits). Subsequent reaction of the distal 5cNO population with a second (proximal) NO generates a transient dinitrosyl. Assuming that the dinitrosyl species exhibit trends in distal off rates similar to those of the 6cNO species (Fig. 3), our data strongly support a balance of affinities mechanism where a high distal *k*_6off_ relative to proximal *k*_off_ in the dinitrosyl precursor traps the proximal 5cNO product in wt AXCP (and also in L16F). In variants where distal 6cNO is trapped (L16A, -V, -I), the values of *k*_6off_ are much smaller than that of wt AXCP while the proximal value may reasonably be presumed to be unchanged. In these variants, proximal NO preferentially dissociates from the dinitrosyl complex and the proximal His ligand rebinds to form the experimentally observed 6cNO complex.

### Implications for proximal NO complexes in heme sensor proteins and the engineering of synthetic heme based sensors: a generally applicable ‘balance of affinities’ mechanism

Several heme proteins, including cytochromes c′, exhibit kinetic behaviour consistent with proximal 5cNO formation *via* 6cNO and putative dinitrosyl precursors. We propose that these proteins may share a common kinetic ‘balance of affinities’ mechanism. In each protein, initial binding of NO to the distal heme face causes His ligand dissociation *via* the *trans* effect, followed by the binding of a second NO to form the dinitrosyl intermediate. The balance of affinities determines which of the two NO ligands in the dinitrosyl dissociates from the Fe and hence which adduct is eventually formed. Where the affinity on the proximal side is higher, the proximal 5cNO form predominates, whereas when the distal affinity is higher, rapid His reattachment leads to a distal 6cNO product. Kinetic data have revealed NO-dependent 6cNO → 5cNO conversion (consistent with proximal 5cNO formation *via* a transient dinitrosyl) in sGC, H-NOX from *Vibrio cholera* (Vc H-NOX),^4^ H-NOX from *Clostridium botulinum* (Cb H-NOX),^6^ and in the cardiolipin/cytochrome c complex,^7^ although we note that structural data are not yet available for these complexes. Although *k*_6on_ values for the 6cNO intermediates of VcH–NOX, Cb H–NOX and sGC proteins are relatively high (≥10^8^ M^–1^ s^–1^) and consistent with a low degree of distal steric hindrance, the corresponding distal 6cNO *k*_6off_ values are also relatively high (0.3, 0.012, and 27 s^–1^, respectively), such that the putative dinitrosyl species might foreseeably decompose by preferential release of distal NO in a manner analogous to that proposed for AXCP.

## Conclusions

Our data show that 6cNO binding to the distal heme face of AXCP predominates when residue 16 is small (Ala, Val, Ile), even when significant distortion of the Fe–N–O angle is present and where a flip of the heme 7-propionate has occurred. In contrast, when Leu or Phe are present in the occluding position, the 6cNO distal complex is an intermediate prior to formation of a proximal 5cNO species. Taken together, our data are consistent with a ‘balance of affinities’ mechanism where the distal pocket occluding residue affects the kinetic parameters *k*_6on_ and *k*_6off_ for NO binding to the distal heme face. The ratio of distal and proximal face affinities determines which NO from the transient dinitrosyl [6c-(NO)_2_] intermediate dissociates, leaving either a distal 6cNO complex (L16A, L16V, L16I) or a proximal 5cNO complex (wt AXCP, L16F). Modification of dinitrosyl dynamics represents a novel strategy for modulating heme–NO response by controlling distal *vs.* proximal heme–NO coordination as well as 6cNO *vs.* 5cNO coordination geometry.

## Methods

### Construction of variants and protein preparation

The preparation of recombinant wt and L16A AXCP were described previously.^13,15^ To generate the L16V, L16I & L16F variants, a modified Quikchange site directed mutagenesis method was applied to the *AXCP* gene in plasmid pet26b(+) using primers shown in ESI.† Protein expression, purification and crystallisation were as described previously^15^ as were procedures to remove any endogenously bound gas ligands. To reduce crystals to the ferrous state they were transferred into ∼2 mL of deoxygenated buffer containing 100 mM ascorbate in a supasealed glass vial for ∼3 h. To generate the NO-bound state, 10 μL of 80 mM stock of the NO donor compound proliNONOate was injected through the supaseal. Crystals were transferred using a cryoloop into cryoprotectant comprising 40% sucrose, 2.4 M ammonium sulfate, 100 mM HEPES pH 7.5 for ∼10 s before transfer into liquid nitrogen.

### X-ray data collection and processing

All crystallographic and single crystal spectroscopic data were measured at the Swiss Light Source, beamline X10SA. X-ray diffraction data were measured using a Pilatus 6M-F detector and processed using XDS.^30^ Data reduction and refinement were carried out in the CCP4i suite using AIMLESS^31^ and REFMAC5^32^ with the most appropriate AXCP structure from the PDB chosen as the starting model. Between cycles of refinement the structures were rebuilt in Coot.^33^ The structures were validated using the JCSG QC server and Molprobity.^34^ On convergence of Refmac5 refinement, structures with resolution 1.25 Å or higher were further refined in Shelxl^35^ to obtain estimated standard uncertainties for bond lengths and angles. *F*_o_ – *F*_c_ omit maps were generated to guide and validate ligand modelling in structures where NO was observed in two alternate positions with partial occupancy (Fig. S5†). Coordinates and structure factors have been deposited in the RCSB Protein Data Bank. Data collection, processing and refinement statistics are shown in (Table S2†).

SCRR spectra were measured from the crystals used for structure determination with the MS3 on-axis microspectrophotometer at beamline X10SA^36^ with a 405.4 nm excitation laser. Spectra were measured prior to and following X-ray data collection to check for changes to the sample caused by the excitation laser or X-ray radiolysis. Raman shifts were calibrated using cyclohexane or paracetamol as a reference. The laser powers at the sample position were selected to be below the threshold of laser-induced photo-reduction and were in the range of 0.88 to 4.30 mW. The SLS-APE toolbox^37^ was used to analyse all SCRR data.

### Resonance Raman spectroscopy

Ferrous L16 variants and their complexes with NO were prepared in an anaerobic glove box. Protein was reduced to the ferrous state with excess sodium dithionite. To prepare RR samples of gaseous heme complexes, excess dithionite was removed using a minispin desalting column (Zeba filter, Pierce), followed by introduction of gas into the headspace of septum-sealed capillaries using a gas-tight Hamilton syringe. The identity of RR samples was verified by UV-vis spectroscopy before and after exposure to the laser beam using a modified Cary 50 spectrophotometer. RR spectra were recorded on a custom McPherson 2061/207 spectrograph (set to 0.67 m) equipped with a Princeton Instruments liquid N_2_-cooled (LN-1100PB) CCD detector. Excitation wavelengths were provided by the 406.7 nm and 413.1 nm lines of a Kr ion laser and the 441.6 nm line of a He–Cd laser. Rayleigh scattering was attenuated using supernotch filters (Kaiser) or long-pass filters (RazorEdge, Semrock). RR spectra of frozen samples, maintained at 100 K with a liquid nitrogen cold finger, were obtained using a ∼150° backscattering geometry and laser powers of 5–25 mW (at the sample). A 90° scattering geometry was used for RR spectra of room temperature samples. RR spectra were typically measured for periods of 2–5 min with indene and aspirin used to calibrate Raman shifts to an accuracy of ±1 cm^–1^.

### Stopped-flow kinetics of NO binding and release

A SX-20 UV-visible stopped-flow spectrophotometer (Applied Photophysics) with a diode-array detector was used to record the reaction of ferrous protein with NO at 25 °C. Spectra were obtained from 360 to 700 nm with a dead-time of ∼1.3 ms. A 40-fold excess of sodium dithionite solution (∼200 μM) was used to prepare the ferrous proteins in degassed buffer (50 mM CHES and 100 mM NaCl, pH 8.9) to match previous experiments.^13^ The NO donor proliNONOate (dissolved in 1 mL of 25 mM NaOH) with a stock concentration of ∼80 mM was diluted into the degassed buffer (pH 8.9) yielding the desired concentrations of free NO.

The concentrations of dissolved NO were maintained at a ∼10-fold excess over the heme binding sites (∼5 μM after mixing) to ensure pseudo-first order conditions. Ferrous protein solutions were mixed with NO-containing buffer in the range of 50–1850 μM (after mixing). Reactions were monitored using monochromatic light at 393, 416 and 436 nm using a photomultiplier detector and from 360–700 nm (0–500 s) using a photodiode array detector. Pseudo-first-order rate constant at each [NO] was determined by fitting exponential time courses using a least-squares fitting method and plotted against the [NO] to yield the second-order-rate constant. The global analysis of multi-wavelength kinetic data was carried out using the Pro-Kineticist software package (Applied Photophysics).

The release of NO from L16V and L16I AXCP was initiated by reacting the nitrosyl complex with a solution of 14–59 mM sodium dithionite in the presence of ∼0.5 mM CO in an anaerobic cuvette. Time-resolved UV-vis absorption spectra, recorded at 25 °C using a Cary 60 spectrophotometer, were used to monitor the rate of disappearance of the 6cNO complex *via* the appearance of 6cCO absorbance features (*λ*_max_ 418 nm). Rate constants for heme–NO release (*k*_6off_) obtained from exponential fits of the 418 nm absorbance time course were insensitive to variations in dithionite concentration.

### Time-resolved infra-red spectroscopy

Time-resolved infra-red (TRIR) experiments were carried out as described previously^28^ using the ULTRA instrument, Central Laser Facility, Rutherford Appleton Laboratory. wt AXCP and the distal L16I and -V variants (5 μM) were prepared anaerobically in a glove box. The ferrous states were achieved by the addition of sodium dithionite (few grains) and the NO-bound states were achieved by the addition of 1 mg proliNONOate powder followed by an incubation of ∼1 hour at 20 °C. Samples were contained in a cell with CaF_2_ windows and a 75 μm pathlength. The cell was rastered to avoid photo-bleaching. The laser excitation wavelength was 532 nm and data were collected between time delays of 0.0005–1 ns within a spectral window of 1300–1800 cm^–1^ using two overlapping 128 pixel detectors. The resolution of the instrument is ∼3 cm^–1^ per pixel and pixel to wavenumber was calibrated using polystyrene. In-house Ultraview (version 3) software was used to process the data and the kinetic parameters were fitted using Origin Pro.
