# Supplementary material for: Engineering proximal vs. distal heme–NO coordination via dinitrosyl dynamics: implications for NO sensor design
Source: Chem Sci. 2016 Nov 16;8(3):1986–94. doi: 10.1039/c6sc04190f (PMC5390784; doi:10.1039/c6sc04190f)
Supplement: Supplementary file 1 [file SC-008-C6SC04190F-s001.pdf]

Electronic Supplementary Material (ESI) for Chemical Science.
This journal is © The Royal Society of Chemistry 2016

Supporting Information

Kekilli et al

Primers for generation of AXCP variants

L16V_F (CGCCAGTCGGCG**GTG**ACGCTGATGG)
L16V_R (CCATCAGCGT**CAC**CGCCGACTGGCG)
L16I_F (CCGCCAGTCGGCG**ATA**ACGCTGATGGCCT)
L16I_R (AGGCCATCAGCGT**TAT**CGCCGACTGGCGG).
L16F_F (GTACCGCCAGTCGGCG**TTC**ACGCTGATGGCCTCGCAC)
L16F_R (GTG CGAGGCCATCAGCGT**GAA**CGCCGACTGGCGGTAC)

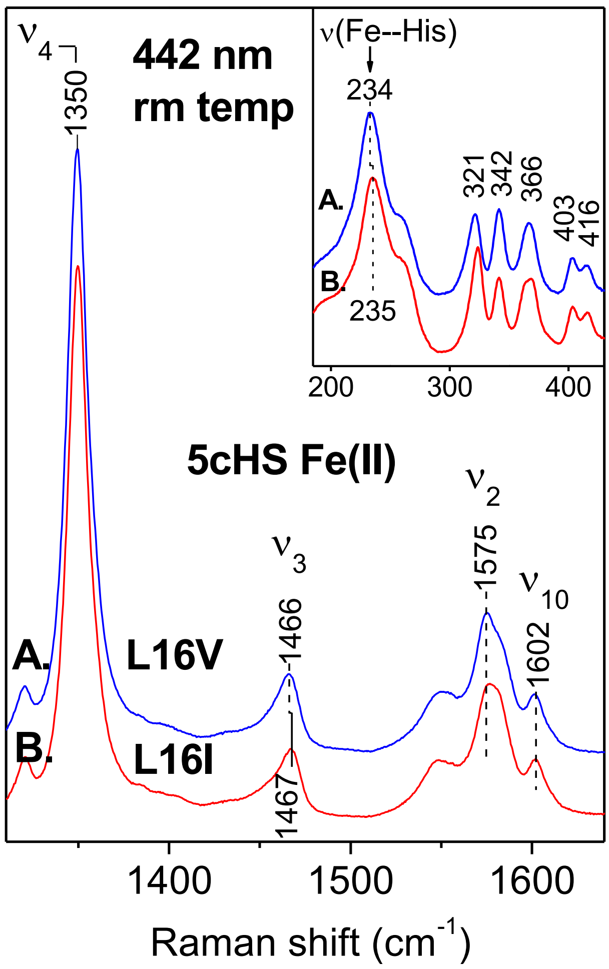

Figure S1. Ferrous RR spectra of (A) L16V and (B) L16I AXCP variants obtained with 442 nm excitation at room temperature. Porphyrin marker bands are characteristic of 5cHS Fe(II) heme. Inset shows the ν(Fe–His) mode in the low-frequency region.

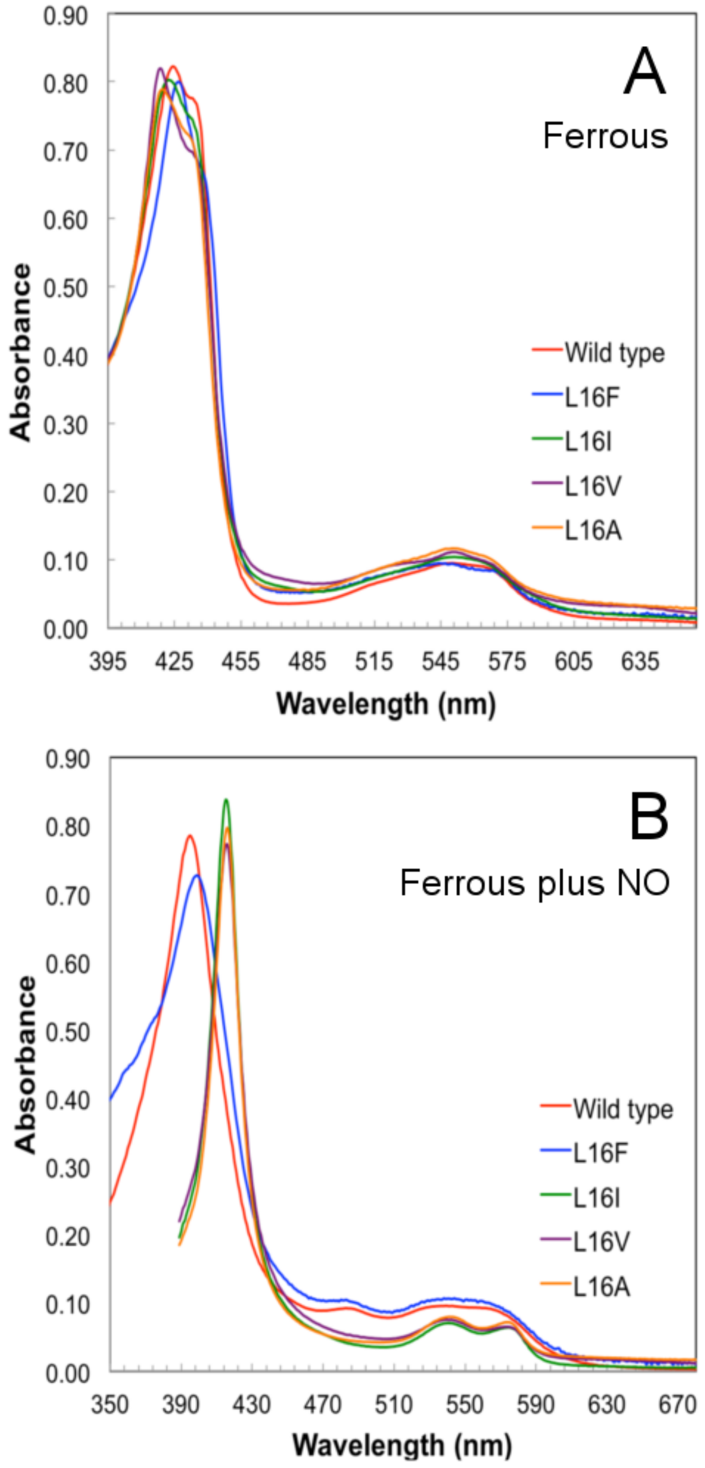

Figure S2. UV-visible absorption spectra of (A) ferrous and (B) ferrous NO-bound wt AXCP and variants.

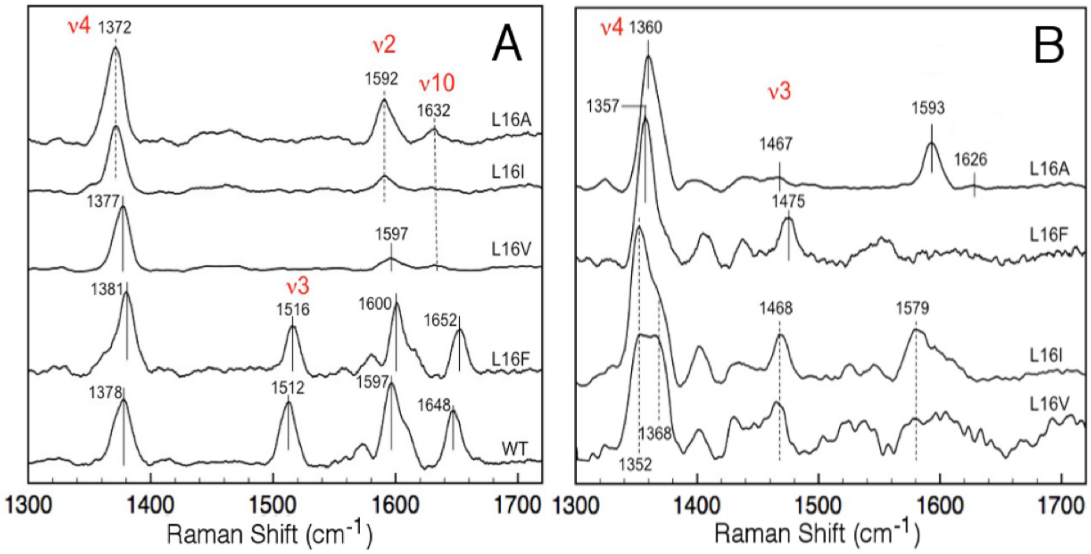

Figure S3. High-frequency single crystal resonance Raman spectra acquired using an excitation wavelength of 405.4 nm at 100K for (A) ferrous-nitrosyl complexes of wt AXCP and variants. The porphyrin marker bands of L16A, L16I and L16V are characteristic of 6cNO and L16F and wt AXCP of 5cNO complexes. (B) High-frequency single crystal resonance Raman spectra of ferrous crystals. Porphyrin marker bands of L16F and L16I are characteristic of 5cHS Fe(II) heme. The L16V variant shows only partial reduction (indicated by the presence of the 1368 cm^{-1} band attributed to ferric protein). The L16A data is consistent with structural data and indicates 6cLS heme, a water molecule occupies the vacant 6th coordination site of the heme Fe in the crystal.

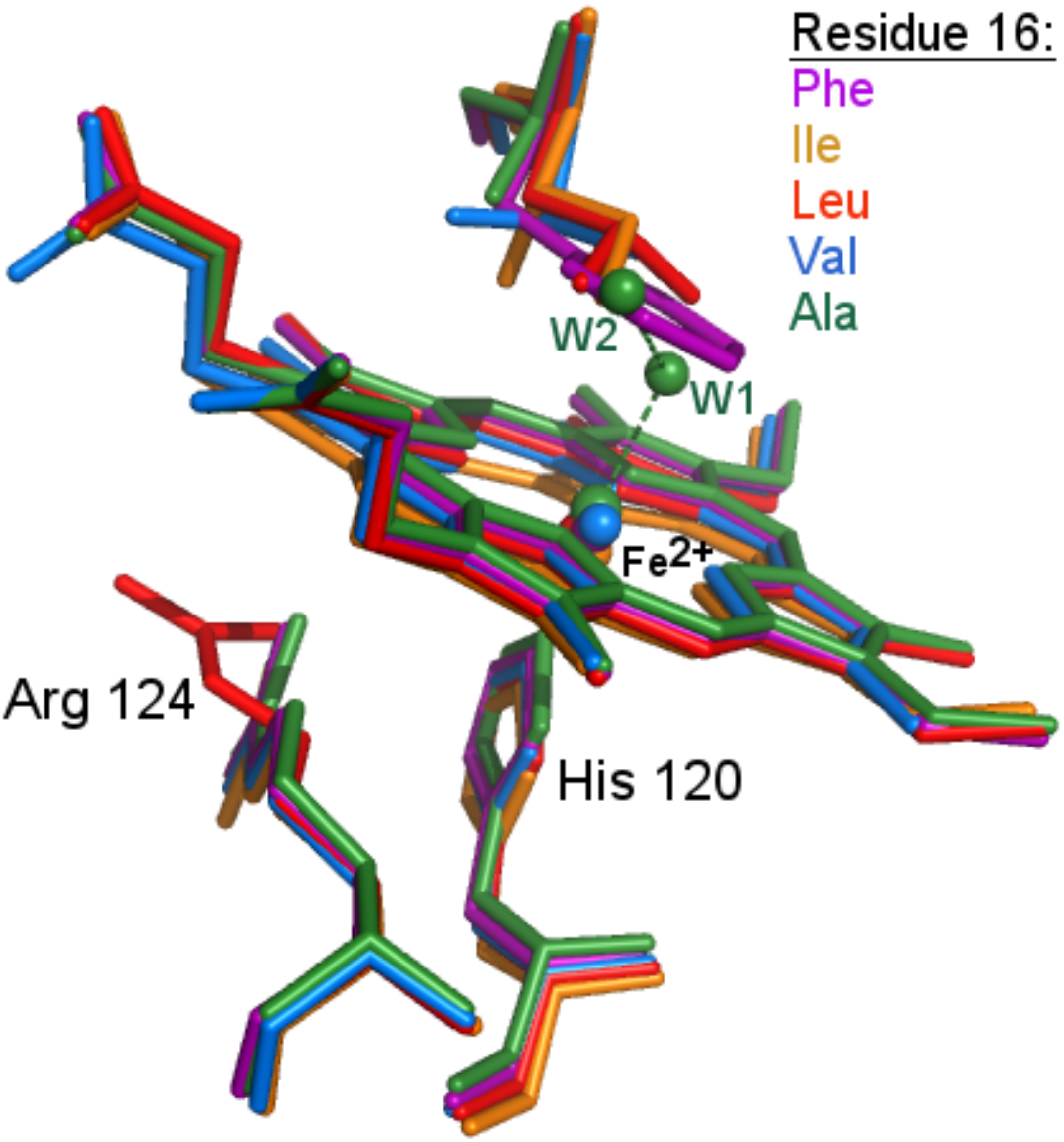

Figure S4. Superposition of the heme environments in ferrous wt AXCP (red), L16A (green), L16V (blue), L16I (orange) and L16F (purple). In ferrous L16A, a water molecule is observed ligated to the distal heme face. This water molecule is not stabilized by hydrogen bonding to any protein residues, but instead forms a hydrogen bond to a second water molecule some 3.0 Å away in the distal pocket. This second water molecule is also bound to the carbonyl of Arg12 (~3.12 Å away). Observation of water molecules in the hydrophobic distal pocket is unusual and may result from the larger distal cavity created by the L16A mutation. Note that in the structure of chemically-reduced ferrous wt AXCP, a minor population (occupancy 0.2) of Arg 124 adopts a position parallel to the heme plane, consistent with a minor population of ferric protein in the crystal.

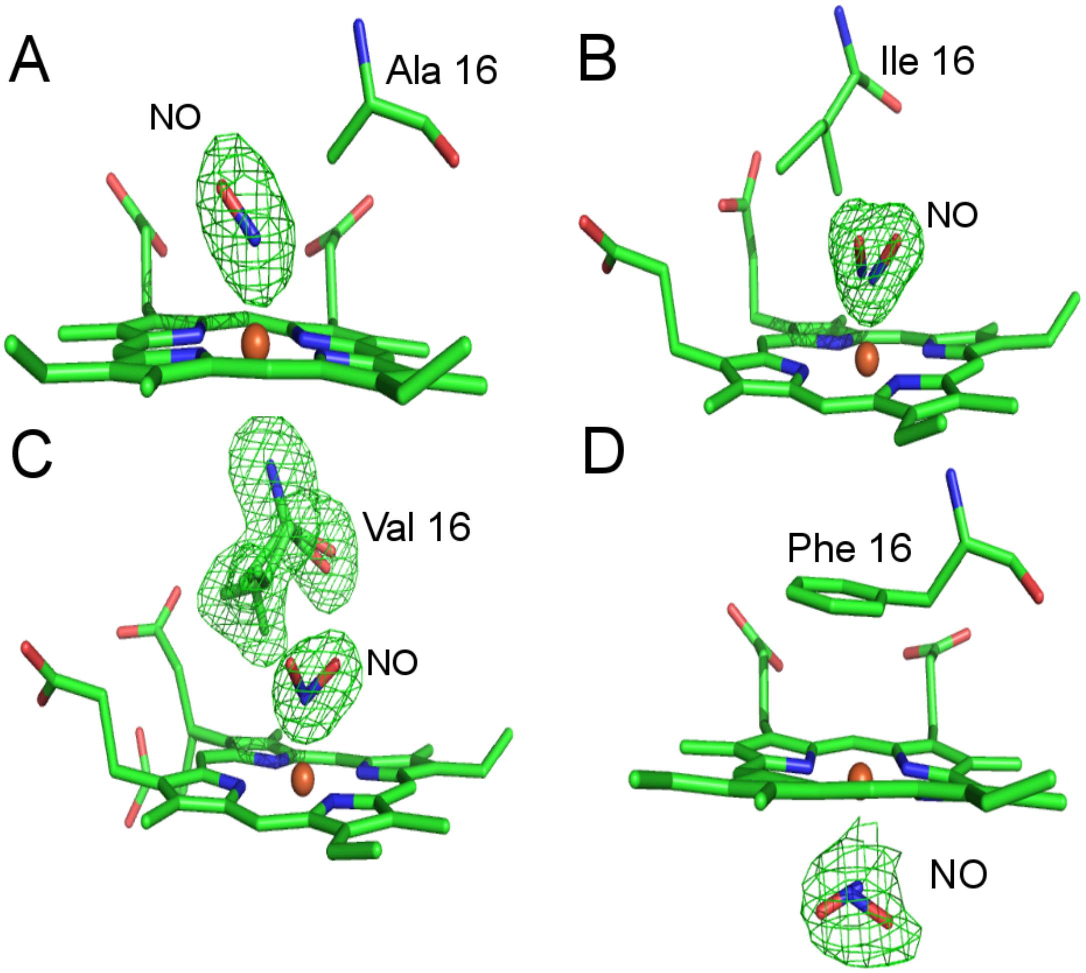

Figure S5. *Fo-Fc* omit maps, contoured at 3σ**,** for the NO complexes of (a) L16A, (b) L16I, (c) L16V and (d) L16F. In (c) the omit map also covers the double conformation of residue Val16.

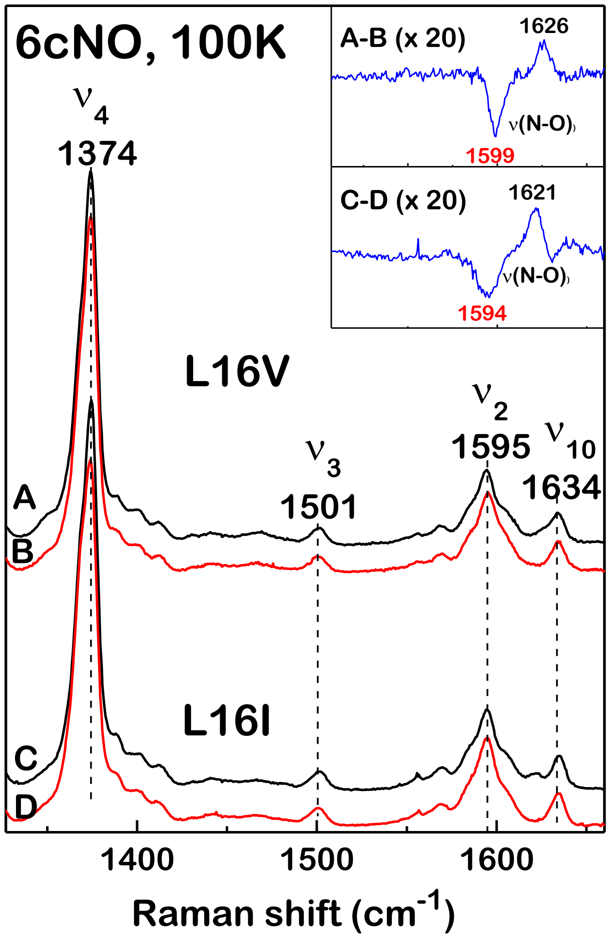

Figure S6. High frequency RR spectra of 6cNO L16V AXCP at 100 K prepared with ^{14}NO (A) and ^{15}NO (B); and L16I AXCP prepared with ^{14}NO (C) and ^{15}NO (D). Assignments of ν(N–O) are based on isotopic substitution difference spectra (A-B) and (C-D).

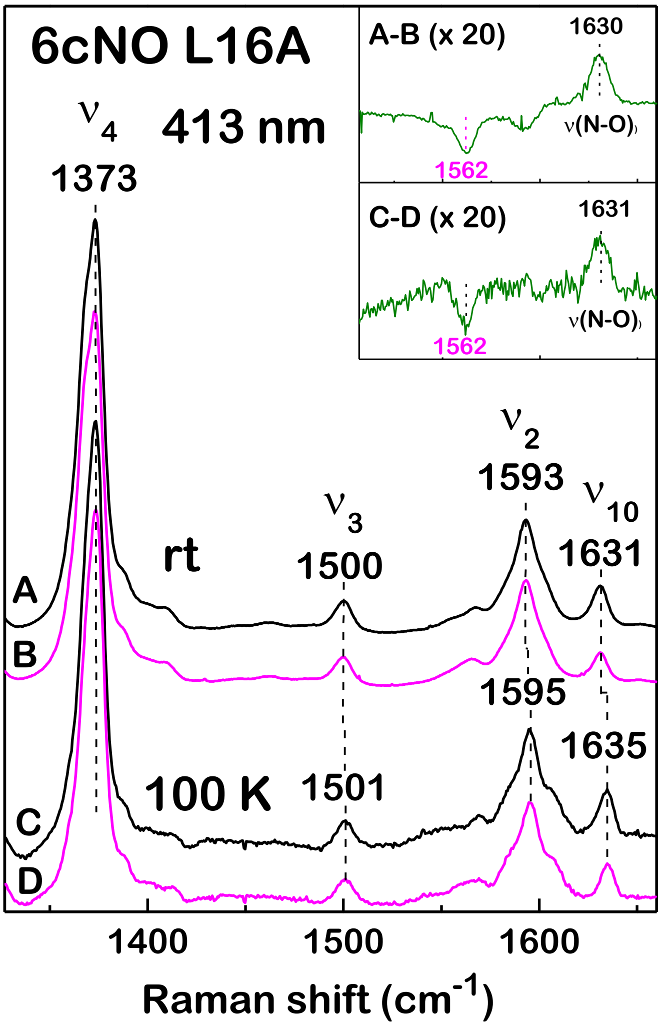

Figure S7. High-frequency RR spectra of 6cNO L16A AXCP obtained with 413 nm excitation. Measurements were performed at room temperature with $^{14}N^{16}O$ (A) and $^{15}N^{18}O$ (B) and at 100 K with $^{14}N^{16}O$ (C) and $^{15}N^{18}O$ (D). Assignments of ν(NO) are based on isotope difference spectra, (A-B) and (C-D).

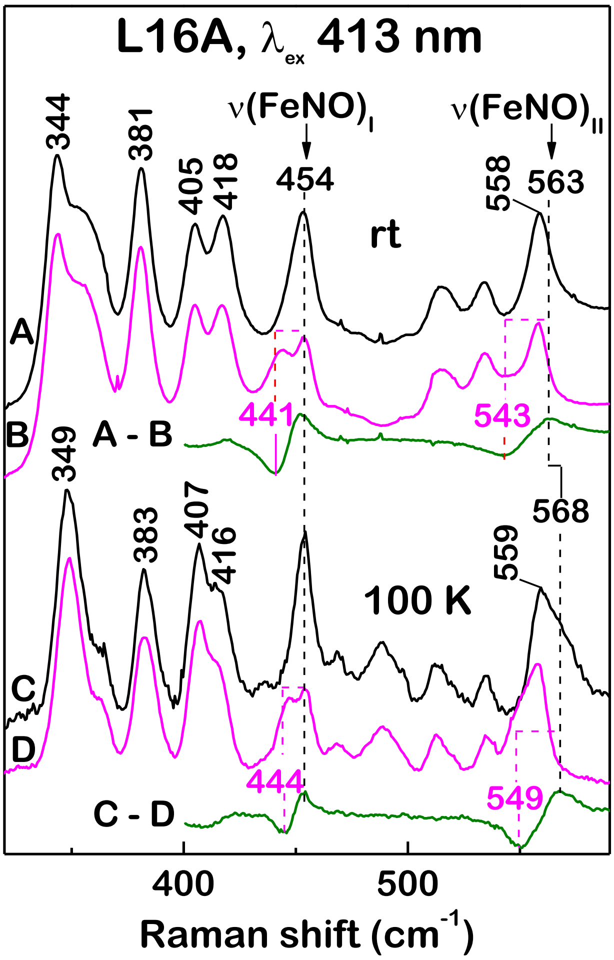

Figure S8. Low-frequency RR spectra of 6cNO L16A AXCP obtained with 413 nm excitation. Measurements were performed at room temperature with $^{14}N^{16}O$ (A) and $^{15}N^{18}O$ (B) and at 100 K with $^{14}N^{16}O$ (C) and $^{15}N^{18}O$ (D). Assignments of ν(FeNO)$_I$ and ν(FeNO)$_{II}$ are based on isotope difference spectra, (A-B) and (C-D).

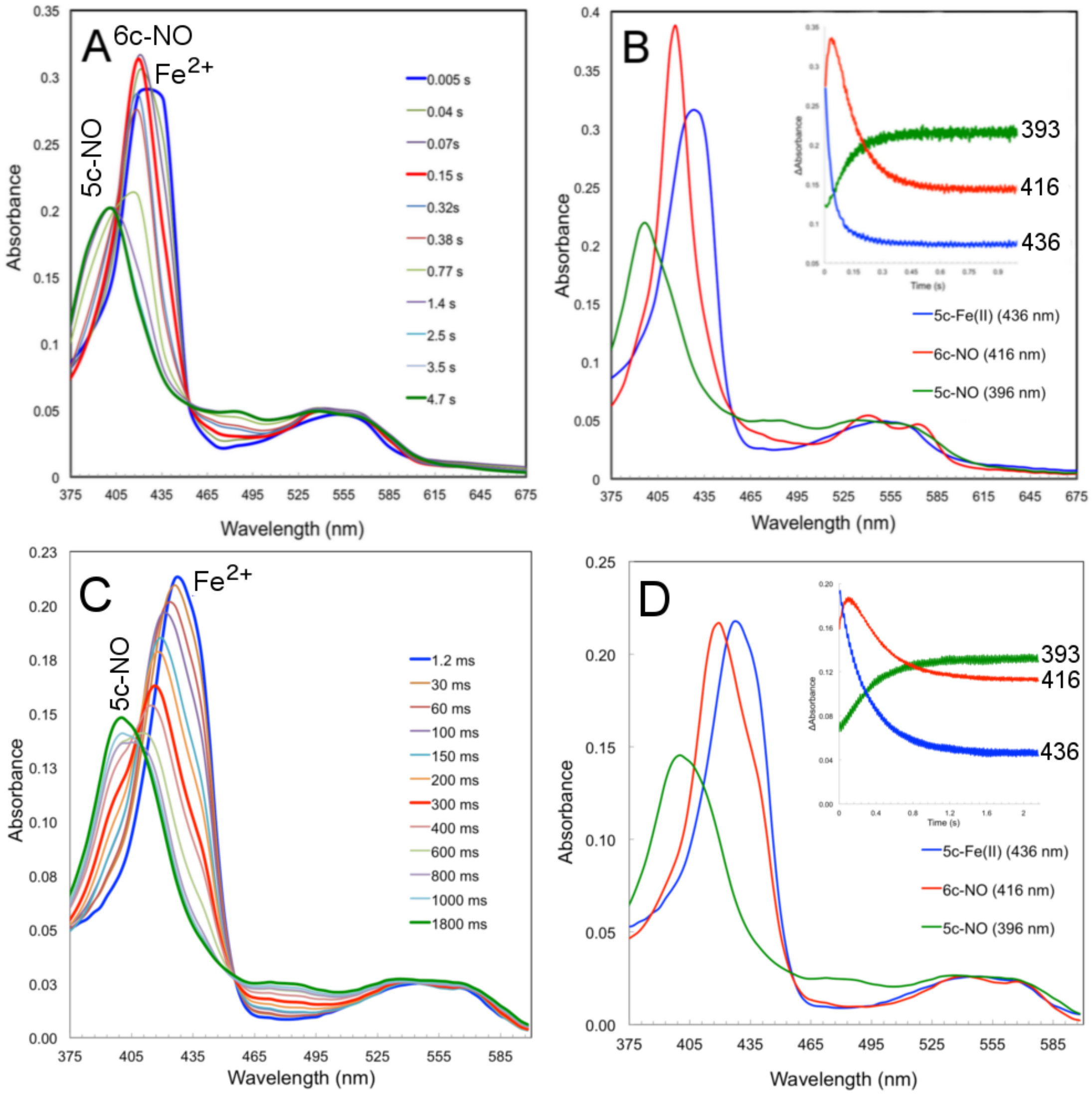

Figure S9. Stopped-flow kinetic data for the reaction of wt AXCP and L16F variant with NO at 25°C in 50 mM CHES and 100 mM NaCl buffer, pH 8.9. (A) Time-dependent spectra for wt AXCP showing the optical transitions recorded from 0.005 to 4.7 s. (B) Extracted spectra of 5c-Fe^{2+}, 6cNO and 5cNO species following global fitting of the data to a sequential A→B→C mechanism. The time-courses (inset) illustrate a rapid first phase and a slower second-phase. (C-D) equivalent data for the L16F variant.

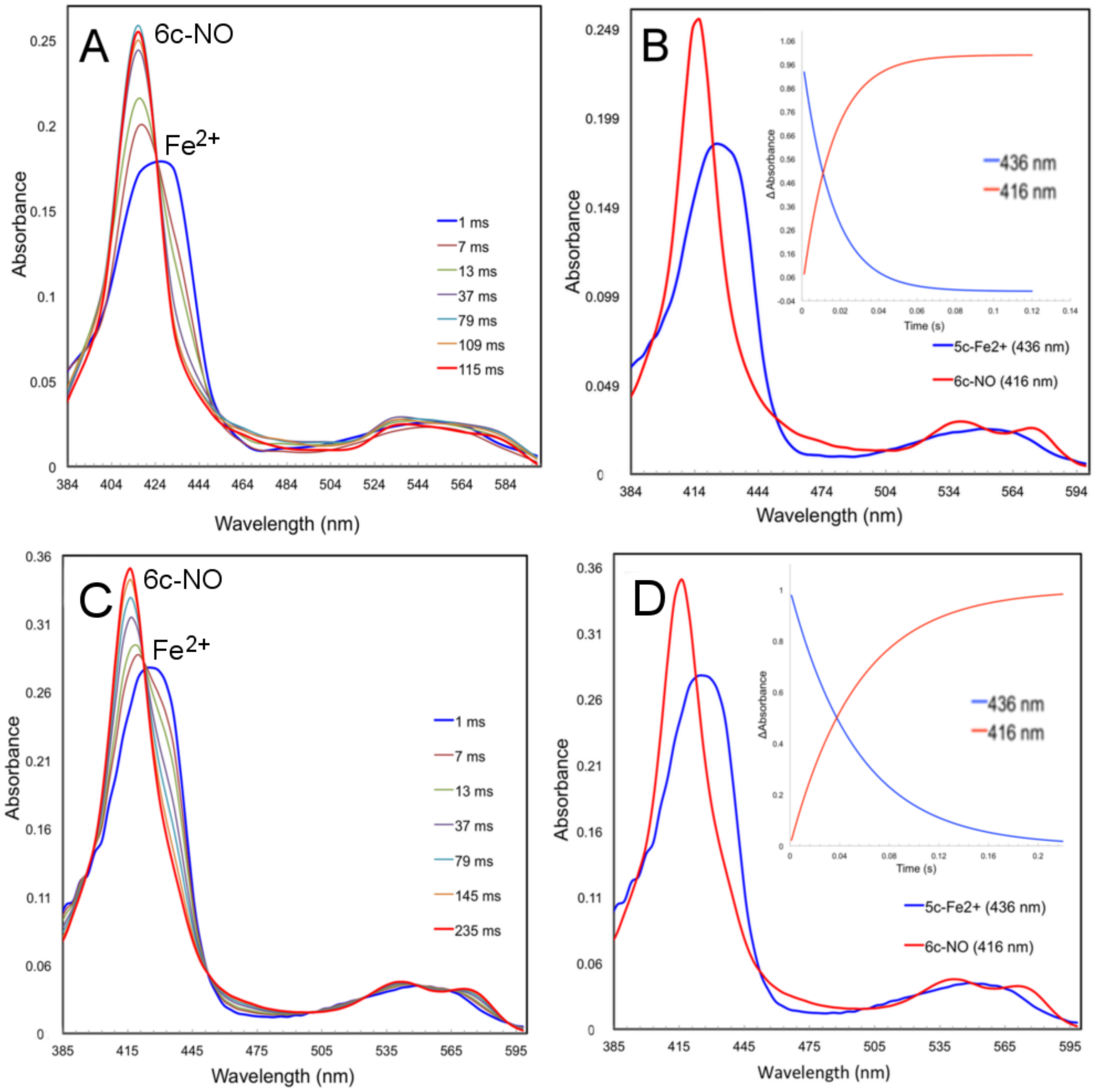

Figure S10. UV-visible stopped-flow kinetic data for the reaction of L16I and L16V variants with NO at 25°C in 50 mM CHES and 100 mM NaCl buffer, pH 8.9. (A) Time-dependent spectra of L16V with optical transitions recorded from 1 to 115 ms showing the build-up of absorption at 416 nm at the expense of the absorption peak at 436 nm. (B) Extracted spectra of 5c-Fe^{2+} and 6cNO species following global fitting of the data to a A→B mechanism. The time-dependent populations are shown in the inset. (C) Time-dependent spectra of L16I with optical transitions recorded from 1 to 235 ms. (D) Extracted spectra of 5c-Fe^{2+} and 6cNO species following global fitting of the data to a A→B mechanism with the time-dependent populations shown in the inset.

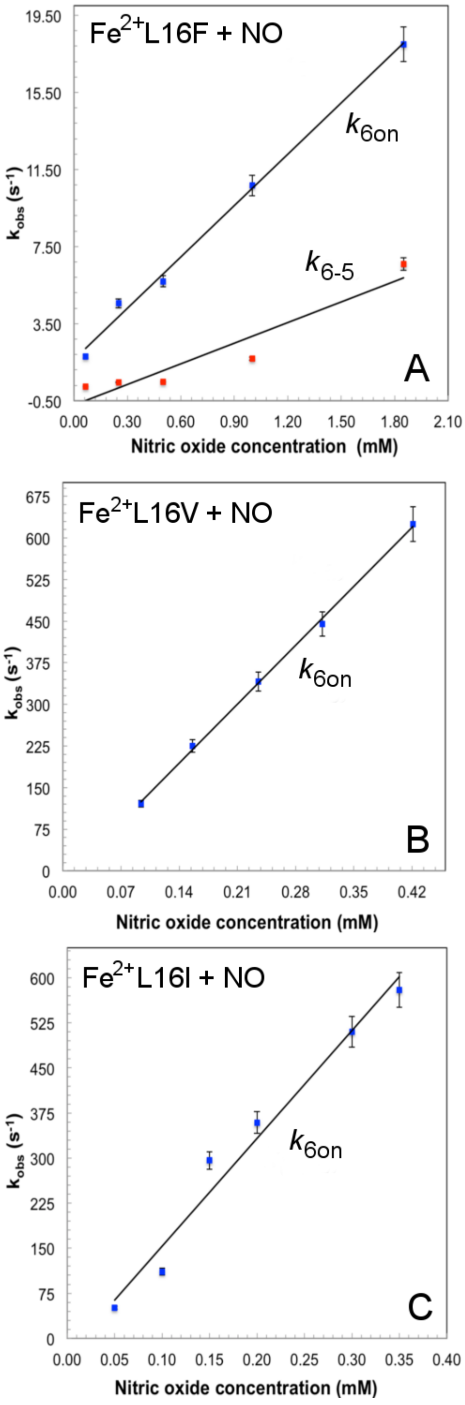

Figure S11. UV-visible stopped-flow data for the reaction of ferrous distal variants with NO. Dependence of k_{obs} on the concentration of NO showing the second-order rate constants for NO binding (k_{6on}) and the formation of the 5c-NO complex ($k_{6\text{-}5}$) for the L16F variant (A). The second-order rate constants for 6c-NO binding (k_{6on}) in the L16V (B) and the L16I variant (C).

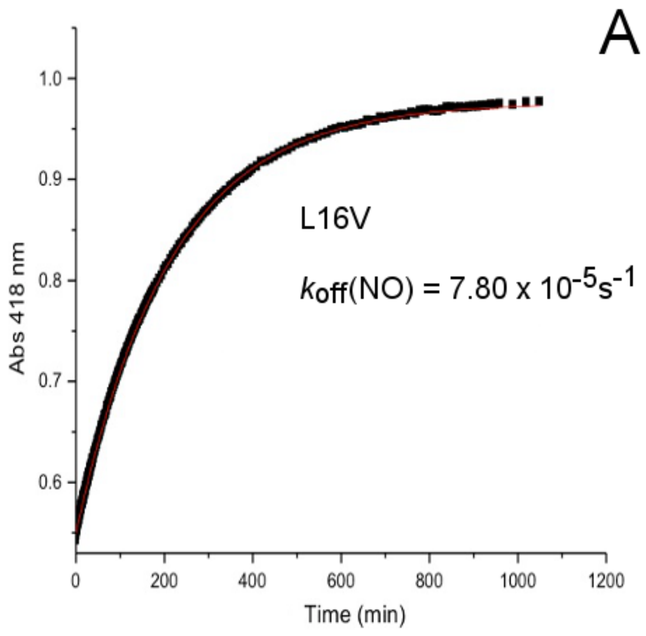

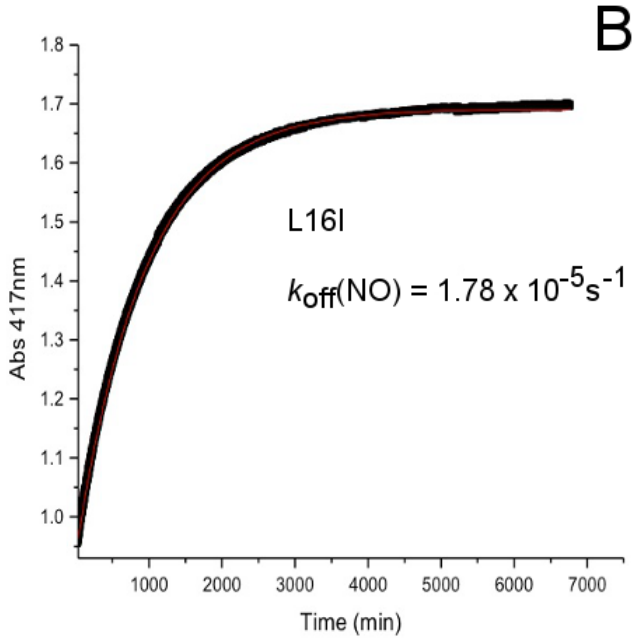

Figure S12. Time trace at 418 nm for the release of NO from 6cNO L16V in the presence of 59 mM dithionite and 1 mM CO (A) and time trace at 417 nm for the release of NO from 6cNO L16I in the presence of 24 mM sodium dithionite and 1 mM CO (B). Overlaid and mostly obscured are single-exponential fits.

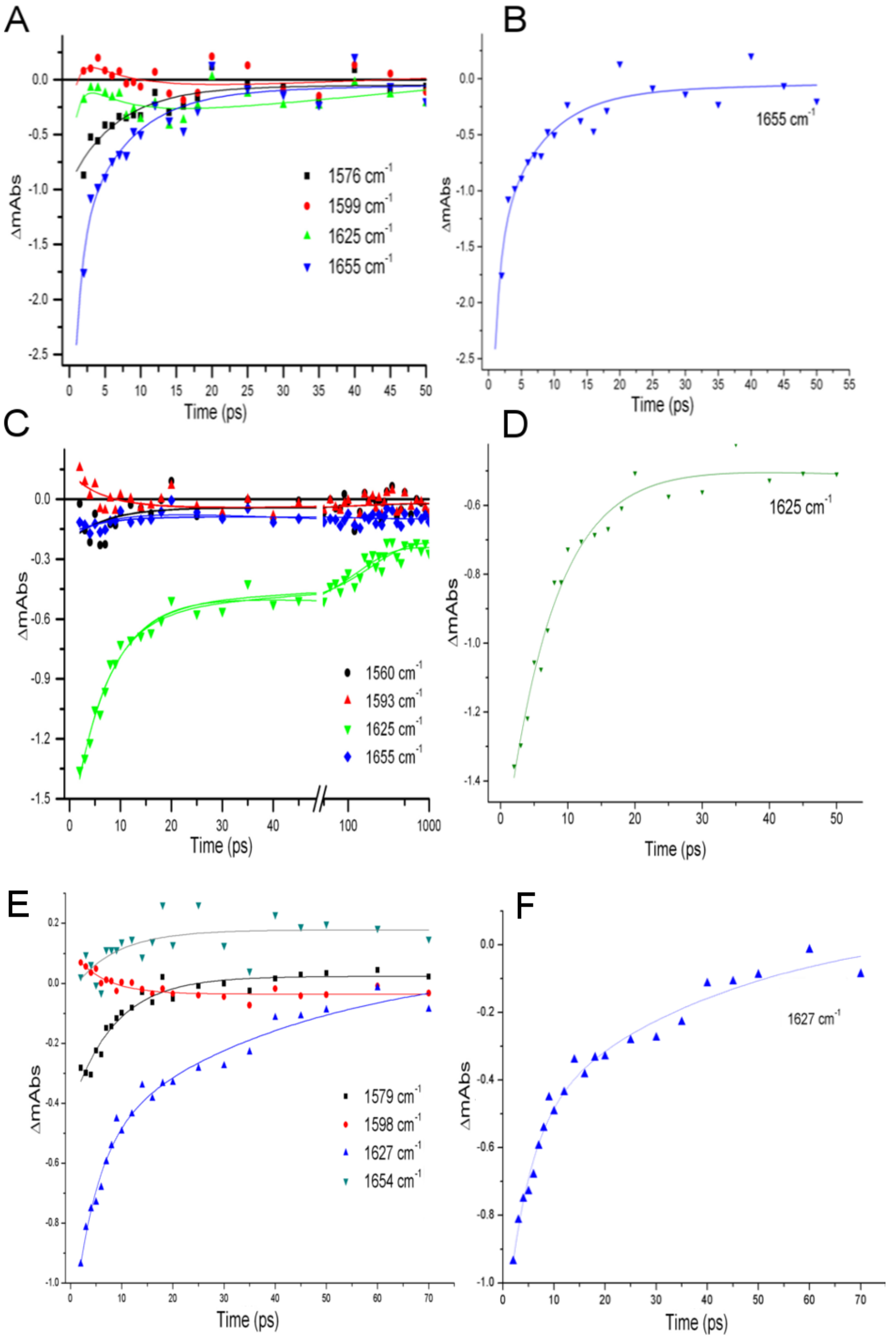

Figure S13. Kinetic decay of wt AXCP and the L16V and –I distal variants. (A) The decay of wt AXCP was fitted to the sum of three exponentials using the wavenumbers at 1576, 1599, 1625 and 1655 cm^{-1}. (B) The kinetic decay at 1655 cm^{-1} for wt AXCP. (C) The decay for L16V was fitted to the sum of two exponentials using the wavenumbers at 1560, 1593, 1625 and 1655 cm^{-1}. (D) The kinetic decay at 1625 cm^{-1} for the L16V variant. (E) The decay for L16I was fitted to the sum of two exponentials using the wavenumbers at 1579, 1598, 1627 and 1654 cm^{-1}. (F) The kinetic decay at 1627 cm^{-1} for the L16I variant.

Table S1. Effect of distal Leu16 mutations on the RR Frequencies (cm^{-1}) of ferrous AXCP. *sh*; shoulder, *nd*; not determined, rt; room temperature, [c] this work.

Fe(II) AXCP	State	T (K)	λ_{ex} (nm)	spin state	ν_4	ν_3	ν_2	ν_{10}	ν(Fe–His)	ref
L16A	solution	rt	413, 442	5cHS	1350	1467	1577	1603	230	[c]
	crystal	*100*	*405.4*	*6cLS*	*1360*		*1593*		*nd*	[c]
L16V	solution	rt	442	5cHS	1350	1466	1575	1602	234	[c]
	crystal	*100*	*405.4*	*5cHS*	*1352 (1368 sh)*	*1468*				[c]
L16I	solution	rt	442	5cHS	1350	1467	1577	1602	235	[c]
	crystal	*100*	*405.4*	*5cHS*	*1352 (1368 sh)*	*1468*	*1579*		*nd*	[c]
wt	solution	rt	413, 442	5cHS	1351	1469	1577	1603	231	16
	solution	90	413, 442	5cHS	1353	1471	1579	1607	*nd*	17
	crystal	*100*	*405.4*	*5cHS*	*1350*	*1467*	*1582*	*1602*	*nd*	15

Table S2. Data collection and refinement parameters for AXCP structures. All data were measured at SLS beamline X10SA using a Pilatus 6M-F detector. Note that estimated standard uncertainty values are given for structures where the final refinement was carried out in Refmac5 and not where SHELXL was used.

Structure	L16A	L16A	L16V	L16V	L16I	L16I	L16F	L16F
State	Ferrous	NO	Ferrous	NO	Ferrous	NO	Ferrous	NO
Wavelength (Å)	0.9	0.9	1.0	1.0	0.9	1.0	0.9	0.9
Resolution (Å)	1.25	1.55	1.25	1.38	1.12	1.13	1.25	1.70
Unique reflections	42926	21801	43208	32339	61939	57236	43297	17754
Completeness (%)	98.9 (98.4)	98.4 (98.4)	100 (99.8)	100 (100)	98.7(94.8)	97.4 (96.6)	100 (100)	99.9 (99.9)
R_{merge} (%)	0.037(0.884)	0.073(0.760)	0.032(0.770)	0.051(0.744)	0.064(0.652)	0.030(0.599)	0.059(0.684)	0.059(0.832)
Mn(I/sd)	22.8 (2.1)	11.9 (2.0)	18.0 (2.1)	15.4 (2.0)	9.6 (1.5)	24.2 (2.5)	11.4 (2.2)	12.7 (2.0)
Redundancy	6.3 (6.2)	6.3 (6.5)	5.0 (4.6)	7.6 (6.8)	5.5 (4.5)	6.2 (6.1)	6.2 (5.8)	6.2 (6.5)
Wilson B-factor (Å^2)	13.7	18.3	15.6	18.2	11.4	12.2	14.0	30.6
R_{cryst} (%)	0.149	0.171	0.173	0.163	0.153	0.161	0.146	0.212
R_{free} (%)	0.191	0.214	0.215	0.186	0.181	0.165	0.179	0.258
ESU based on ML (Å)	-	0.069	-	0.042	-	-	-	0.120
RMSD Bond lengths (Å)	0.017	0.017	0.012	0.017	0.015	0.014	0.014	0.017
RMSD. Bond angles(°)	2.4	2.2	2.5	2.5	2.2	2.6	2.3	2.3
Ramachandran favoured (%)	98.5	97.8	98.6	97.8	98.5	96.4	98.5	98.4
PDB accession code	5JT4	5JLI	5JP7	5JRA	5JVE	5JUA	5JSL	5JS5

Table S3. Structural parameters from crystal structures of ferrous AXCP variants.

Residue 16	Resolution (Å)	Fe-His (Å)	Fe-water (Å)
Ala	1.25	2.03 (±0.02)	2.17
Val	1.25	2.10 (±0.02)	-
Ile	1.12	2.09 (±0.01)	-
Leu[a]	1.22	2.14	-
Phe	1.25	2.14 (±0.02)	-

All values are from this work, except *a*[1]. Values in parentheses are estimated standard deviations determined from Shelx full matrix refinement.

ESI References

1 1. D. Kekilli, F. S. N. Dworkowski, G. Pompidor, M. R. Fuchs, C. R. Andrew, S. Antonyuk, R. W. Strange, R. R. Eady, S. S. Hasnain and M. A. Hough, *Acta Cryst.,* 2014*,* **D70**, 1289.

2 E. M. Garton, D. A. Pixton, C. A. Peterson, R. R. Eady, S. S. Hasnain and C. R. Andrew, *J. Am. Chem. Soc.*, 2012, **134**, 1461.

3 C. R. Andrew, E. L. Green, D. M. Lawson and R. R. Eady, *Biochemistry,* 2001, **40**, 4115.

4 C. R. Andrew, S. J. George, D. M. Lawson and R. R. Eady, *Biochemistry* 2002, **41**, 2353.
